# Speckle tracking echocardiography analyses of myocardial contraction efficiency predict response for cardiac resynchronization therapy

**DOI:** 10.1186/s12947-018-0148-5

**Published:** 2018-11-19

**Authors:** Zibire Fulati, Yang Liu, Ning Sun, Yu Kang, Yangang Su, Haiyan Chen, Xianhong Shu

**Affiliations:** 1 Department of Echocardiography, Shanghai Institute of Medical Imaging; Shanghai Institute of Cardiovascular Diseases, Zhongshan Hospital, Fudan University, 180 Fenglin Road, Shanghai, 200032 China; 2 Department of Physiology and Pathophysiology, School of Basic Medical Sciences, Fudan University, Shanghai, China; 3 Department of Cardiology, School of Medicine, Renji Hospital, Shanghai Jiaotong University, Shanghai, China; 4 Department of Cardiology; Shanghai Institute of Cardiovascular Diseases, Zhongshan Hospital, Fudan University, Shanghai, China

**Keywords:** Speckle tracking echocardiography, Strain, Contraction efficiency, Cardiac resynchronization therapy, Prognosis

## Abstract

**Background:**

In patients with left ventricular (LV) dysssynchrony, contraction that doesn’t fall into ejection period (LVEj) results in a waste of energy due to inappropriate contraction timing, which was now widely treated by cardiac resynchronization therapy(CRT). Myocardial Contraction Efficiency was defined as the ratio of Efficient Contraction Time (ECTR) and amplitude of efficient contraction (ECR) during LVEj against that in the entire cardiac cycle. This study prospectively investigated whether efficiency indexes could predict CRT outcome.

**Methods:**

Our prospective pilot study including 70 CRT candidates, parameters of myocardial contraction timing and contractility were measured by speckle tracking echocardiography (STE) and efficiency indexes were calculated accordingly at baseline and at 6-month follow-up. Primary outcome events were predefined as death or HF hospitalization, and secondary outcome events were defined as all-cause death during the follow-up. 16-segement Standard deviation of time to onset strain (TTO-16SD) and time to peak strain (TTP-16SD) were included as the dyssynchrony indexes.

**Results:**

According to LV end systolic volume (LVESV) and LV eject fraction(LVEF) values at 6-month follow-up, subjects were classified into responder and non-responder groups, ECR (OR 0.87, 95%CI 0.78–0.97, *P* < 0.05) and maximum longitudinal strain (MLS) (OR 2.22, 95%CI 1.36–3.61, *P* < 0.01) were the two independent predictors for CRT response, Both TTO-16SD and TTP-16SD failed to predict outcome. Patients with poorer myocardial contraction efficiency and better contractility are more likely to benefit from CRT.

**Conclusions:**

STE can evaluate left ventricular contraction efficiency and contractility to predict CRT response. When analyzing myocardial strain by STE, contraction during LVEj should be highlighted.

## Introduction

Randomized controlled trials have demonstrated that cardiac resynchronization therapy (CRT) is an effective therapy for patients suffering from heart failure refractory to medical therapy with widened electrocardiographic QRS complexes and reduced ejection fraction (EF) [1, 2]. Current guidelines [3] recommend LBBB with QRS duration of ≥150 ms by electrocardiography (ECG) for CRT. However, more than 30% of patients do not benefit, and some may even experience worsening from CRT [4].

**Fig. 1:**
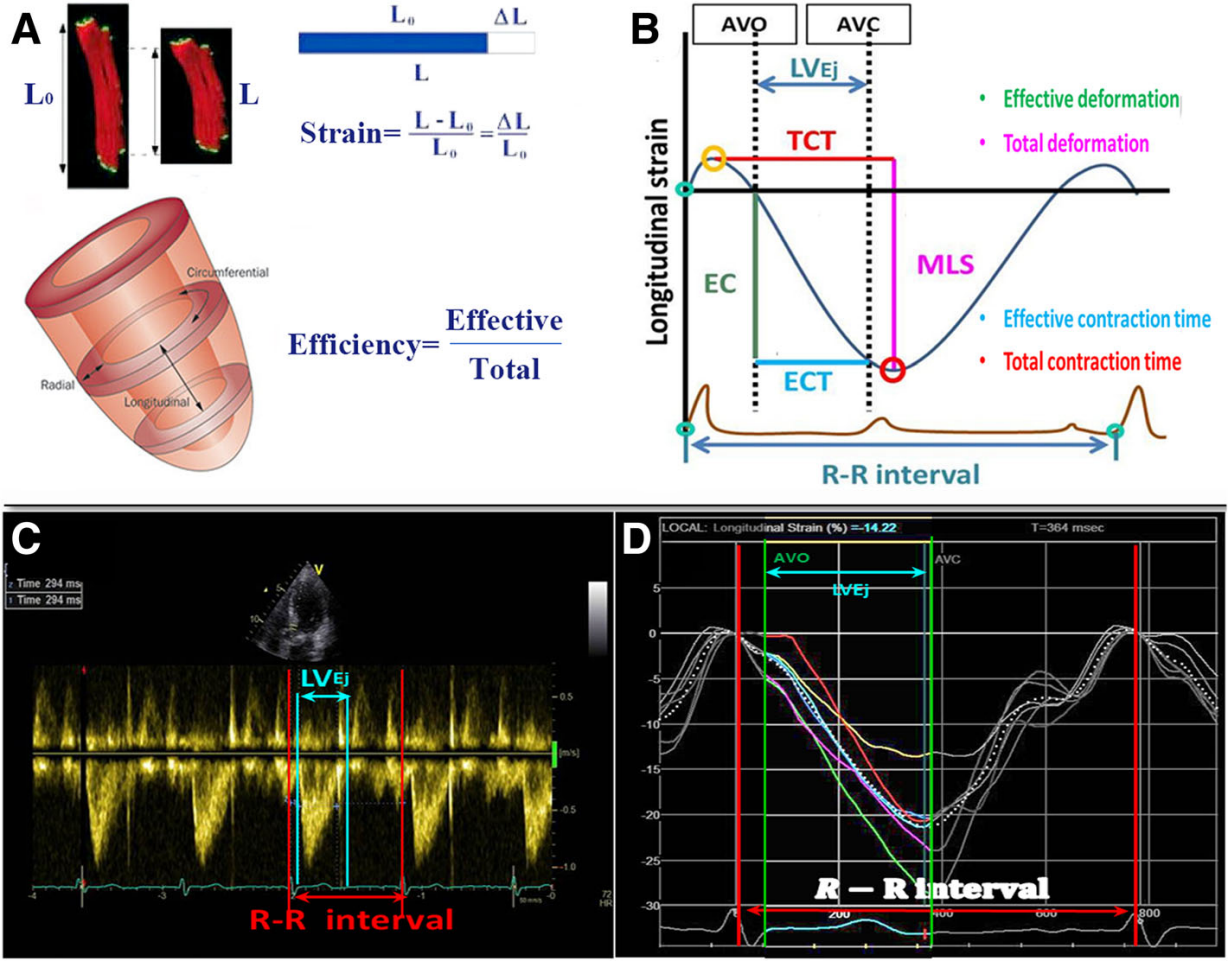
Measurement of amplitude and timing of the effective contraction and the total contraction. **a** illustrates the definition of Strain and the concept of Myocardial Contraction Efficiency which was put forward for the first time in the current study**. b** Longitudinal myocardial shortening within left ventricular ejection (LV_Ej_) and throughout a beat were defined as effective contraction (EC, green line) and maximum longitudinal strain (MLS, amaranth line), respectively. Time spent for EC and MLS were defined as effective contraction time (ECT, blue line) and total contraction time (TCT, red line), respectively. **c** shows the pulsed-wave Doppler ultrasound in the LV outflow tract. The horizontal axis represents velocity (m/s) and the vertical axis represents time (ms). EKG was used as the reference of cardiac cycle timing (green curve). Timing of aortic valve opening and closure were measured at the beginning and the end of the envelope and LV ejection period was then determined (period between blue lines). **d** show the longitudinal strain curve by 2D speckle tracking for a healthy control. The Y axis displays longitudinal strain (%) and the X axis displays time according to EKG (ms). The reference line (the left red line) was placed at the beginning of the QRS complex when the longitudinal strain was defined as zero. Aortic valve closure (AVC) and opening (AVO) were defined according to (**c**). The majority of contraction occurs within LV_Ej_ in healthy control. AVO: aortic valve opening; AVC: aortic valve closure; LV_Ej_: left ventricle ejection period;EC: effective contraction; MLS: maximum longitudinal strain; ECT: effective contraction time; TCT: total contraction time

Observational studies suggested that mechanical rather than electrical dyssynchrony can predict the CRT outcome [5]. Nevertheless, the results of the PROSPECT (Predictors of Response to CRT) trials and [6] the Echo-CRT (Echocardiography Guided Cardiac Resynchronization Therapy) trials [7] make echocardiography dyssynchrony index a controversial tool in selecting patients for CRT. Of note, both studies mainly adopted dyssynchrony indexes of time to peak systolic velocities by Tissue Doppler Imaging (TDI). However, time to peak contraction alone was not sufficient to determine patients’ response to CRT, and time to onset of contraction can actually affect patients’ responsiveness to CRT as well. Further, information about the nature of the cardiac wall deformation, such as viability or scarring that can influence the contraction amplitude, are also key factors influencing the outcomes [8–13].

speckle tracking echocardiography (STE) is widely used to assess both timing and amplitude of myocardial contraction and has been proved to be superior to TDI in ultrasonic angle dependency, signal-to-noise ratio, and its ability to detect viable versus scarred myocardium (Fig. 1a) [14–17]. However, in most studies using STE [18–20], period of interest was defined according to the beginning of QRS complex on the ECG and the aortic valve closure (AVC) on pulsed-wave Doppler imaging of left ventricular outflow tract. Since CRT candidates suffer electrical-mechanical coupling disorder, it is inappropriate to determine the beginning of mechanical contraction based on the onset of electrical activation. Furthermore, only when aortic valve is opening, can myocardial contraction contribute to left ventricle output. Thus, we used aortic valve opening (AVO) instead of the beginning of QRS in STE and highlighted the mechanical contraction performance from AVO to AVC, i.e. the left ventricular ejection period (LVEj) (Fig. 1b, c, d). Since the aortic valve keeps open during LV_Ej_, the myocardial contraction will directly contribute to changes of LV volume and can be regarded as an effective contraction. The ratio of time and amplitude of contraction during LV_Ej_ against those throughout the cardiac cycle reflects the contraction efficiency. The current study hypothesized that low contraction efficiency indicated a high contractile reserve. Patients with low contraction efficiencies are more likely to respond to CRT.

## Material and method

### Study population

The prospective pilot and observational study involved patients referred to our department for CRT implantation from January 2013 to December 2015. The inclusion criteria were as follows: (1) congestive heart failure patients remained symptomatic in New York Heart Association class (NYHA) III or stable class IV, despite optimal pharmacological therapy; (2) left ventricular ejection fraction ≤35%; (3) QRS duration ≥120 ms. The exclusion criteria were: (1) atrial fibrillation (AF); (2) previously implanted pacemaker; (3) recent cardiac event (< 3 months after an acute coronary syndrome or cardiac surgery); or (4) poor acoustic window or failure to undergo implantation of a biventricular device due to anatomic constraints. Then, patients with a reduction≥15% in LVESV together with a rise of ≥10% in LVEF at the 6-month follow-up were defined as CRT responders. The remaining ones were considered as non-responders. Primary outcome events were predefined as death or HF hospitalization, and secondary outcome events were defined as all-cause death during the follow-up. Thirty healthy subjects with normal physical and laboratory conditions served as control group.

### Echocardiography

A complete standard echocardiography examination, including gray-scale images for 2D strain analysis (mean frame rate 64 ± 11 frames/s) was performed the day before CRT implantation using a VIVID E9 imaging system (GE-Vingmed, Horton, Norway). Offline analyses were conducted with a standard imaging software (EchoPac BT12 GE-Vingmed). The LV end-systolic volume, LV end-diastolic volume and LVEF were calculated by the biplane Simpson’s rule [21].

### Two dimensional strain echocardiography

Aortic valve opening and closure were defined using a pulsed-wave Doppler imaging in the LV outflow tract with a 2-mm sample volume, both of which were used as the reference points for LV ejection period (LV_Ej_) **(**Fig. 1c). Speckle tracking of apical LV long-axis views was performed as previously described [22]. The endocardial border was traced in end-systole and the automatically generated region of interest was adjusted to exclude the pericardium. In case of poor tracking, the region of interest tracing was readjusted. Segments with persistent inadequate tracking were excluded from analysis. In case of inadequate tracking in two or more segments, the patient was excluded from analysis. All strain analysis was performed blind to the CRT outcome. The average of three measurements of each STE parameters were used for further analysis and the average time needed to calculate STE parameters were also recorded.

### Definition of amplitude and timing of the effective contraction and the total contraction (Fig. 1b, d)

#### Parameters for the assessment of contractile amplitude

**Maximum longitudinal strain (MLS):**

Sum of the absolute value of maximum positive strain and maximum negative strain throughout the whole cardiac cycle.

**Effective Contraction (EC):**

Longitudinal myocardial shortening occurs within left ventricular ejection.

#### Parameters for the assessment of contractile timing

All time-related indexes were corrected by R-R interval and indicated as a percentage of the cardiac cycle to avoid the influences by heart rate variation.

**Time to the onset of contraction (TTO):**

The time interval from the beginning of QRS to the point when a certain myocardial segment starts to contract, followed by strain curve with a declining trend.

**Time to Peak Strain (TTP):**

The time interval between the beginning of QRS and the lowest point of the strain curves.

**Total contractile time (TCT):**

Total time spent when maximum longitudinal strain occurred.

**Effective contractile time (ECT):**

The sum of time spent during LV_Ej_ when longitudinal shortening occurred.

#### Contractile efficiency indexes

Contractile efficiency indexes were evaluated by the comparison of contractile patterns within LV_Ej_ and throughout the whole cardiac cycle.

**Ratio of effective contraction (ECR):**

$$ \mathrm{ECR}=\mathrm{EC}/{\mathrm{MLS}}^{\ast }100\%; $$

**Ratio of effective contractile time (ECTR):**

$$ \mathrm{ECTR}=\mathrm{ECT}/{\mathrm{TCT}}^{\ast }100\%. $$

#### Conntractile dyssynchrony indexes

**Standard deviation of 16 segments’ time to onset of contraction (TTO-16-SD):**

On apical four chamber view (A4C), apical two chamber view (A2C) and apical three chamber view (A3C), TTO results of 6 segments were obtained separately. TTO of apical septum on A4C and apical anterior septum on A3C were averaged to calculate that of apical septum; while TTO of apical inferior wall on A2C and apical posterior wall on A3C were averaged to calculate apical septum and apical inferior, and thus we can get TTO results of 16 segments in all. The standard deviation of the 16 segments’ TTOs was calculated to evaluate the synchronism of the start of contraction.

**Standard deviation of 16 segments’ time to peak strain (TTP-16-SD):**

TTP results of 16 segments was measured and calculated like TTO results. The standard of the 16 segments’ TTPs was calculated to evaluate the synchronism of the end of contraction.

### Intra-observer and inter-observer reproducibility

STE parameters including EC, ECT, ECR, ECTR, MLS, TTO and TTP of thirty randomly selected examinations (20 pre-implant patients and 10 controls) were re-evaluated by the original observer and a second independent observer. The intra-observer and inter-observer reproducibility were evaluated by Bland-Altman method comparison and intra-class correlation coefficient (ICC) [23, 24].

### Statistical analysis

Continuous variables are expressed as mean ± SEM. Baseline categorical data were compared by means of the Fisher’s exact test. The comparison between two groups was performed by unpaired t-test and that among three groups by One-way ANOVA.

Based on the hypothesis that CRT response would be strongly associated with the contraction pattern within left ventricle ejection (evaluated by the parameters including EC, ECT) and its relation to that of the cardiac cycle (evaluated by the parameters including TCT, MLS, ECR and ECTR), bivariable logistic regression was used to testify the ability to predict CRT response. Based on previous reports of the associations between mechanical synchrony and CRT outcomes [25], TTO, TTP and dyssynchrony indexes of them were also analyzed using bivariable logistic regression. Receiver operating curve (ROC) analysis and multinomial logistic regression were also performed to evaluate the accuracy of these variables in predicting CRT response. For all tests, a *p* value < 0.05 was considered significant. All analyses were carried out with use of the SPSS software (version 20.0, SPSS Inc., USA).

## Results

### Baseline characteristics

Of the 81 patients enrolled, 11 patients were excluded because of atrial fibrillation (7%), previously implanted pacemaker (2%), poor image qualities (2%) or unsuccessful implantation due to anatomic constraints (1%). Accordingly, 70 patients with complete baseline data were included. 49 subjects were male and 21 were female with a mean age of (62 ± 10) years. Eight cases suffered from ischemic heart disease. Five cases (7%) died before 6-month follow-up, who were considered as non-responders. The Responder and the Non-responder group showed comparable age, gender composition, QRS duration, etiology, NYHA functional class and LVEF. However, a significantly larger LV volume was observed in the Non-responder group and a significantly higher TTO dyssynchrony index was seen in the responder group (Table 1 shows the baseline characteristics of the Responder and Non-responder groups).

**Table 1:** Baseline Characteristics

Variables	Total Cohort (*n* = 100)			p Value
	Control Group (*n* = 30)	Non-responder Group (*n* = 32)	Responder Group (*n* = 38)	
Age, yrs	46 ± 1	62 ± 2	62.35 ± 1.67	< 0.0001
Female/Male	12/18(1:1.5)	8/24(1:3)	13/25(1:1.9)	0.01
NYHA functional class	/	3.03 ± 0.67	2.85 ± 0.46	0.17
QRS, ms	81 ± 2.52	149 ± 6.37	151 ± 5.26	< 0.0001
Ischemic etiology	/	4(12%)	4(10%)	0.64
LVEF, %	69.45 ± 0.73	26.50 ± 1.91	26.45 ± 1.05	< 0.0001
LVEDV, ml	62.50 ± 1.93	282.30 ± 21.56	203.00 ± 11.30***	< 0.0001
LVESV, ml	18.80 ± 0.61	210.40 ± 20.14	150.20 ± 9.29**	< 0.0001
TTO-16-SD, %	2.85 ± 0.23	9.65 ± 0.85	14.04 ± 2.01*	< 0.0001
TTP-16-SD, %	3.73 ± 0.18	12.45 ± 1.12	16.83 ± 2.90	< 0.0001

*LVEF* Left Ventricle Eject Fraction, *LVEDV* Left ventricle end diastolic volume, *LVESV* Left ventricle end systolic volume, *TTO-16-SD* 16-segement Standard deviation of time to onset strain, *TTP-16-SD* 16-segement Standard deviation of time to peak strain

Compared Responder Group to Non-responder Group: *p < 0.05; **p < 0.01; ***p < 0.001

### Changes of contraction timing

#### Pre-implant (baseline)

**Fig. 2:**
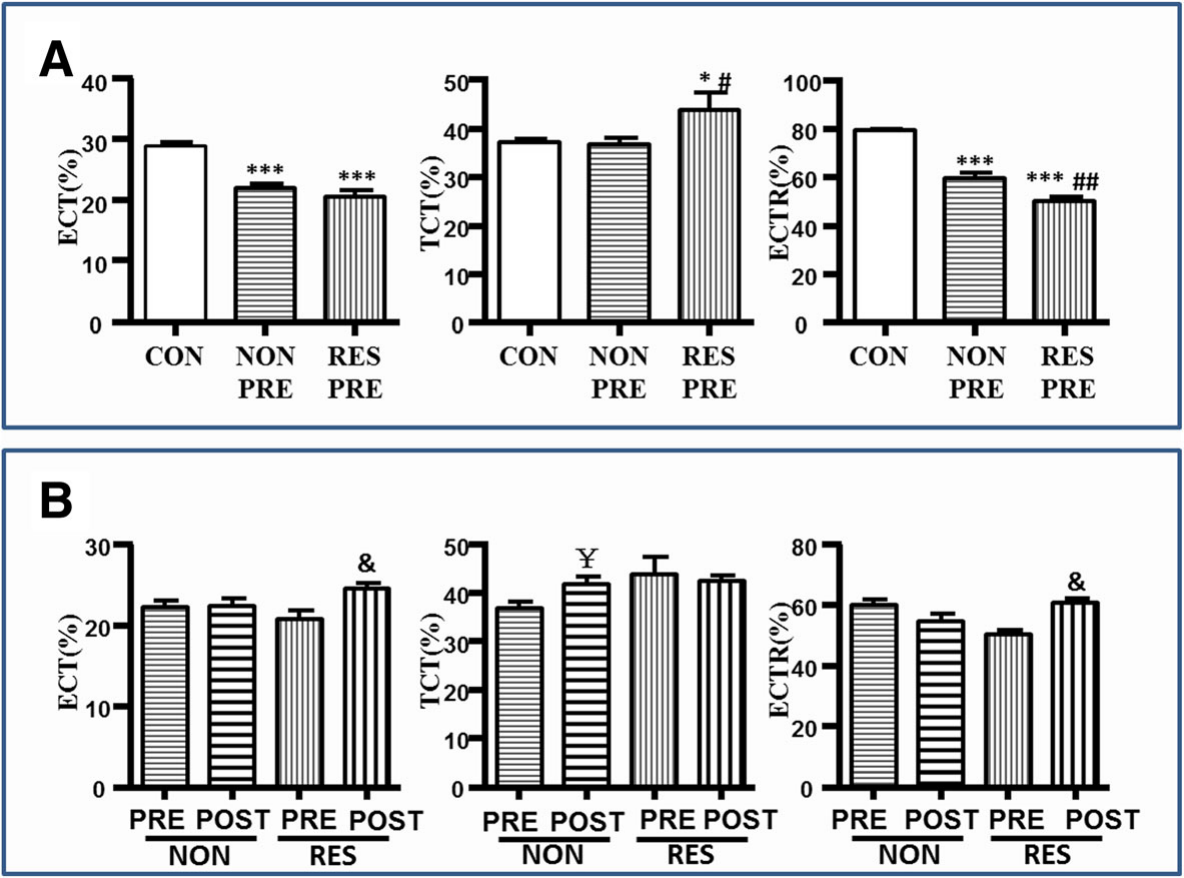
Comparison of contraction timing indexes. **a** Comparison among control group, baseline non-responder group and responder group. (Compared to Control Group: *p < 0.05; **p < 0.005; ***p < 0.0005; Compared to baseline Non-responder Group #*p* < 0.05; ##*p* < 0.005; ###*p* < 0.0005). **b** Comparison between baseline and 6-month follow-up in both patient groups. (Comparison between pre-CRT and post-CRT in Non-responder Group: ¥*p* < 0.05; Comparison between pre-CRT and post-CRT in Responder Group: &*p* < 0.05;) ECT: effective contraction time; TCT: total contraction time; ECTR: effective contraction time ratio

ECT of both Non-responder and Responder groups decreased significantly compared with that of the control group. That is to say the time of contraction during left ventricle ejection period decreased in both patient groups and thus their left ventricles had less time to pump forward blood into the systemic circulation. TCT of the Responder group was longer than that of the Control group. It was very likely a compensation pattern occurred in the Responder group. A longer TCT can to some degree extended ECT. However, a shorter ECT with a longer TCT in the Responder group would inevitably make them exhibiting the worst ECTR which was defined as ECT/TCT. Similarly, the Control group exhibited the best ECTR, followed by that of the Non-responder.Obviously, timing of contractile efficiency deteriorated in both patient groups at baseline, yet the Responder group had an even more energy-consuming contractile pattern. However, as we mentioned when introducing the definitions of efficiency indexes, a poorer ECTR indicates a higher possibility to be improved **(**Fig. 2a, Table 2**)**.

**Table 2:** Compare STE parameters in Baseline and 6-month follow up

	Control Group	Non-responder Group		Responder Group	
	(n = 30)	Baseline (n = 32)	6-month (*n* = 27)	Baseline (n = 38)	6-month (n = 38)
ECT(%)	29.26±0.50^*,#^	22.26±0.82	23.31 ± 6.09	20.86±1.03**	24.62±0.70
TCT(%)	37.26±0.6^*,#^	36.83±1.46^##^	41.93±1.54	43.95±3.58	43.21 ± 5.64
ECTR(%)	79.51±0.69^*,#^	60.00±2.16	59.1 ± 11.88	50.30±1.64**	60.94±1.57
EC(%)	17.09±0.59^*,#^	5.35±0.41	7.82 ± 3.91	5.44±0.31**	9.71±0.53
MLS(%)	18.67±0.58^*,#^	8.11±0.39	11.33 ± 3.92	9.28±0.36**	13.13±0.52
ECR(%)	91.60±0.72^*,#^	62.90±2.53	59.1 ± 11.88	53.69±1.69**	72.16±1.86

*EC* effective contraction, *ECT* effective contractile time, *ECR* the ratio of effective contraction, *ECTR* the ratio of effective contractile time, *MLS* maximum longitudinal strain, *TTO* time to onset strain, *TTP* time to peak strain, *6 month* 6 month follow-up

Compare Non-responder Group versus Control Group at baseline,* *P* < 0.05;

Compare Responder Group versus Control Group at baseline, # *P* < 0.05;Compare Baseline versus 6 month follow up in Non-responder group, ## *P* < 0.05;

Compare Baseline versus 6 month follow up in Responder group, ** *P* < 0.05;

#### Post-implant (6-month follow-up)

Compared to baseline (Pre-implant), ECT and ECTR improved significantly and TCT remained unchanged in the Responder group. The Responder group had a more efficient contractile pattern after CRT. Although the Non-responder group failed to achieve a higher ECTR or a longer ECT, it did start a similar compensation contractile pattern with a longer TCT as the Responder group did at baseline. TCT of the Non-responder extended after CRT. **(**Fig. 2b, Table 2**)**.

### Changes of contraction amplitude and efficiency

#### Pre-implant (baseline)

**Fig. 3:**
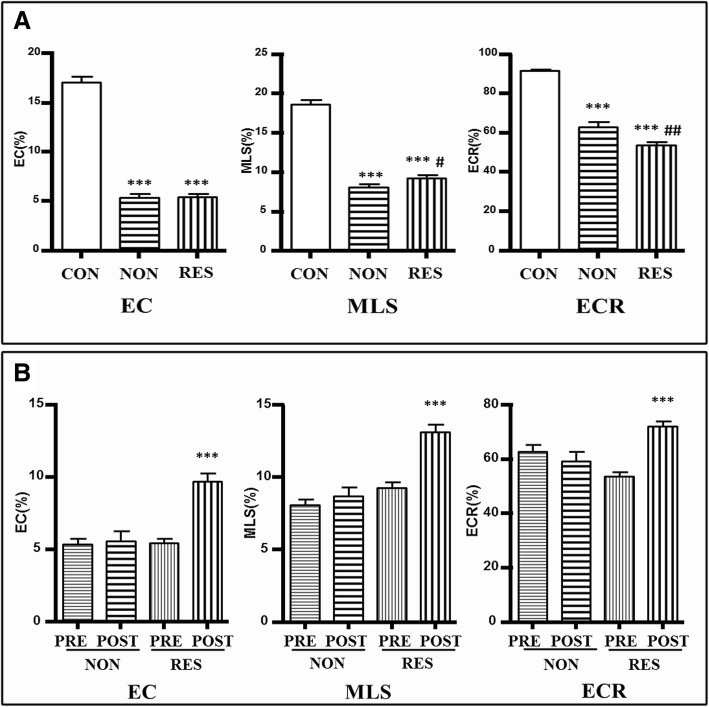
Comparison of contraction amplitude between baseline and 6-month follow-up. **a** EC, MLS and ECR decreased significantly in both patient groups. The responder group had better MLS (p < 0.05) and worse ECR (p < 0.005) than the non-responder group. (Compared to Control Group: *p < 0.05; **p < 0.005; ***p < 0.0005; Compared to baseline Non-responder Group #*p* < 0.05; ##*p* < 0.005; ###*p* < 0.0005.) EC: effective contraction; MLS: maximum longitudinal strain; ECR: effective contraction ratio. **b** All the parameters of contraction amplitude improved in the responder group and unchanged in the non-responder group. (Comparison between pre-CRT and post-CRT in Responder Group: *p < 0.05; **p < 0.005; ***p < 0.0005) EC: effective contraction; MLS: maximum longitudinal strain; ECR: effective contraction ratio

EC of both Non-responder and Responder group decreased significantly than that of the Control group. This may result from the weakened myocardium contractility and the previously stated finding that ECT shortened in both patient groups. Moreover, the Control Group showed the best ECR, followed by those of the Non-responder group, while the Responder group exhibited the worst ECR. This suggested that the low EFs of both patient groups were related not only to the contractility disorder but also to the contractile efficiency. Besides, the Responder group again showed the worst efficiency in contractility, consistent with the performance of its efficiency in contraction timing, enhancing its possibility to be improved. It was interesting for us to find that when most the parameters of mechanical contraction ability and efficiency turned out to be the worst in the Responder group, and it had better MLS than Non-responder group. A better MLS means a better contractility or myocardial vitality, so although the Responder group showed a worse contractile efficiency than the Non-responder group, it had a better baseline contractility or viability. Together with the previous mentioned finding that the Responder group may extend its TCT as compensation to maintain its ECT and ECTR, it is very likely that the Responder group may actually be at an earlier stage of the disease than the Non-responder group (Fig. 3a, Table 2).

#### Post-implant (6-month follow-up)

**Fig. 4:**
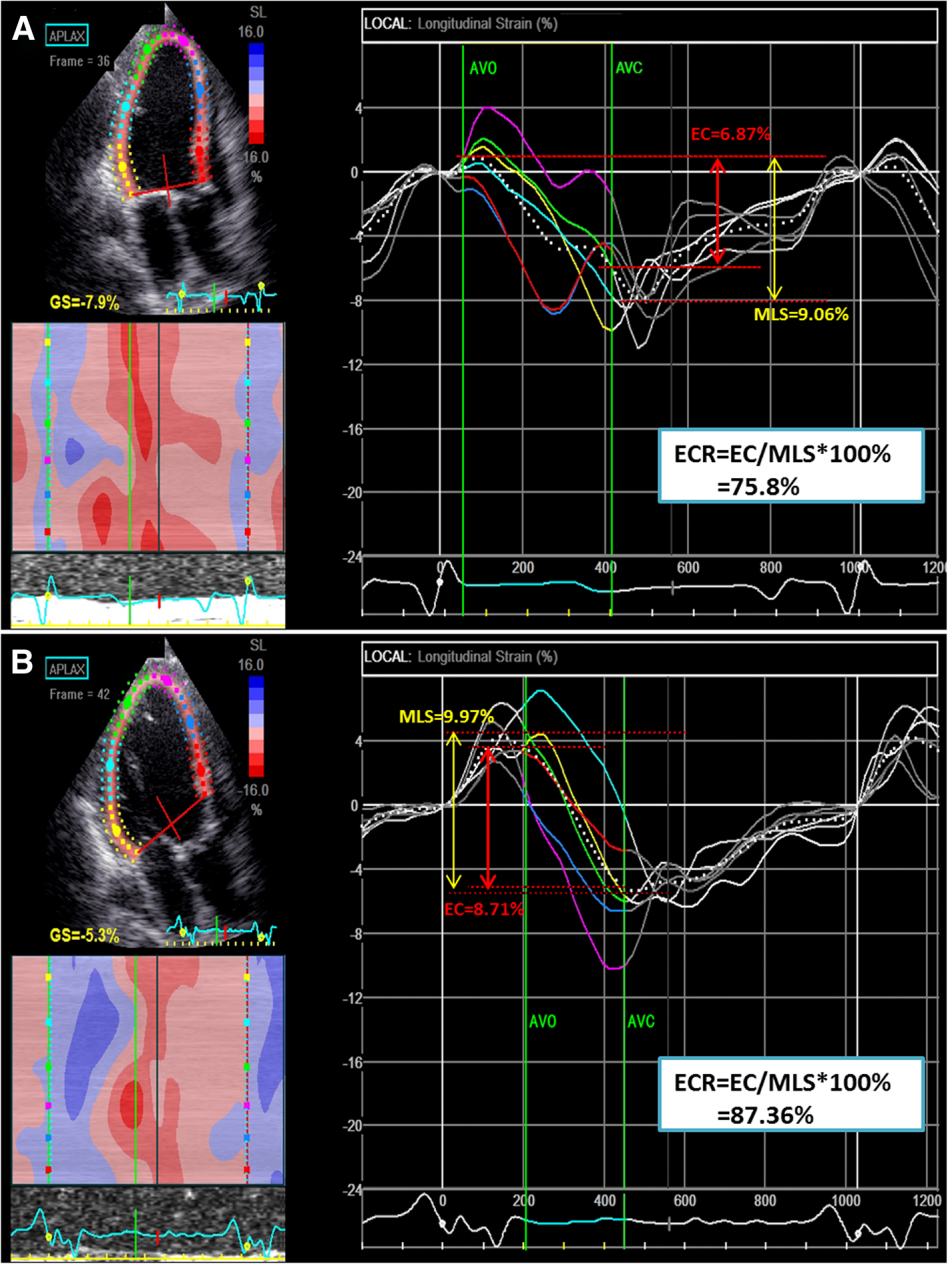
Longitudinal strain curves of a CRT responder. **a** and **b** shows the longitudinal strain curves for a responder at baseline and 6-month follow up respectively. The patient’s LVEF improved from 25 to 47%. QRS was recognized by software as the beginning of systole. Since the negative peak before AVC was automatically recorded as the peak strain, the global strain (GS) by software was − 7.9% at baseline and − 5.3% at follow-up. These findings were contrary to the LVEF improvement. However, after studying the EC within LV_Ej_ and the MLS throughout the cardiac cycle, we found that MLS was slightly improved, while EC and ECR were significantly improved

All parameters of contractility and contractile efficiency including EC, MLS and ECR were improved significantly in Responder Group while remain unchanged in the Non-responder Group (all *p* > 0.05) **(**Figs. 3b and 4, Table 2**)**. CRT can improve the electrical activation sequence of myocardium segments and increase EC through changing the time of the events. An improved MLS may be achieved by the virtuous cycle of the energy-saving contractile in the remaining viable myocardium.

### Predictors for CRT response

Among all parameters, ECTR and ECR exhibited the best AUC in predicting non-response for CRT, while MLS had the best AUC in predicting response **(**Table 3**)**. However, only ECTR and MLS were proved to be the predictors for CRT response by multinomial logistic regression **(**Table 4**)**. ECTR and ECR are the indexes for contraction efficiency. A high ECTR or ECR represents a better contractile efficiency and indicates less potential to be improved. However, ECTR provided nothing but the information of contractile timing and neglected the contractility or the viability. Therefore it turned to be useless in predicting CRT outcome. Although MLS is not an index measured within LV_Ej_, it represents the myocardial maximum reformation ability, or viability.

**Table 3:** ROC analysis for CRT response

Variable	AUC	cut-off	Sensitivity (%)	Specificity (%)
Predictors of non-response				
ECTR	0.682	59.02%	85.0	58.8
ECR	0.724	62.68%	85.0	52.9
Predictor of response				
MLS	0.694	8.81	70.0	76.5

*ECTR* the ratio of effective contractile time, *ECR* the ratio of effective contraction, *MLS* maximum longitudinal strain

**Table 4:** Logistic regression for CRT response

Variable	OR(95% CI)	Wald X^2^	*p* value
Binary logistic regression			
ECTR	0.928(0.887~ 0.972)	10.030	0.002
ECR	0.943(0.904~ 0.983)	7.692	0.006
MLS	1.257(1.014~ 1.557)	4.349	0.037
Multinomial logistic regression (*p* = 0.012)			
ECR	0.867(0.775~ 0.969)	6.264	0.012
MLS	2.219(1.364~ 3.612)	10.299	0.001

*ECTR* the ratio of effective contractile time, *ECR* the ratio of effective contraction, *MLS* maximum longitudinal strain

### Average time for calculating STE parameters

The Average time consumed for calculating EC, ECT, MLS, TTO and TTP were 165 ± 25 s(about 2~ 3 min) while the time spent for ECR and ECTR were about 240 ± 45 s(about 4~ 5 min).

### Intra-observer and inter-observer reproducibility

**Fig. 5:**
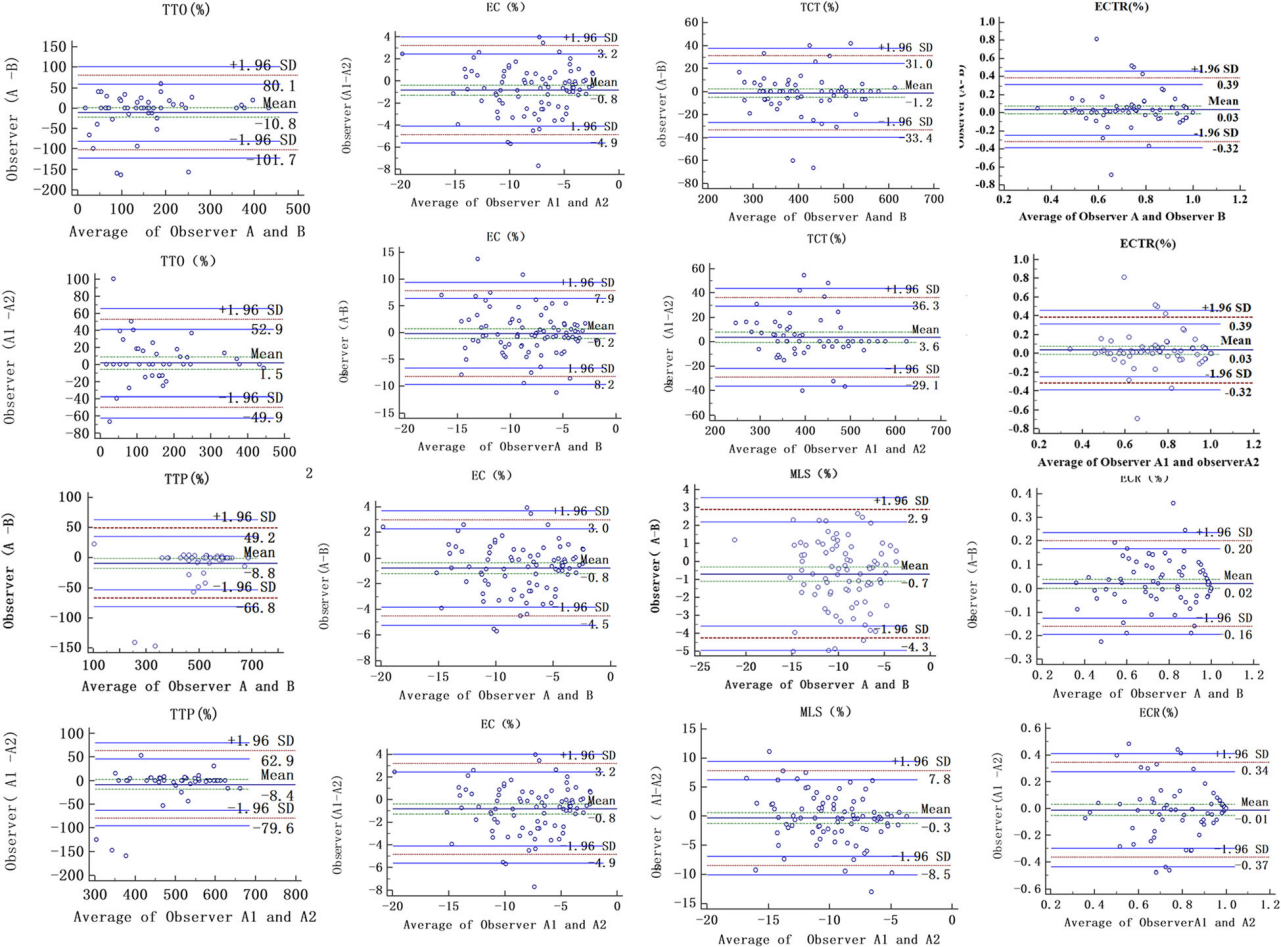
Intra-observer and Inter-observer reproducibility. It shows the inter-observer reproducibility for the value of inter-observer ICC and intra-observer ICC for EC, ECT, ECR, ECTR, MLS, TTO and TTP. It suggested a good reproducibility of all STE parameters. ECT: effective contractile time; EC: effective contraction; ECR: the ratio of effective contraction; ECTR: the ratio of effective contractile time; MLS: maximum longitudinal strain; TTO: time to onset strain; TTP: time to peak strain

The value of inter-observer ICC and intra-observer ICC for EC and ECT, ECR ECTR, MLS, TTO, TTP were analyzed respectively (all *p* < 0.0001**)**, and the result indicated a favorable reproducibility [23, 24] (Fig. 5 and Table 5).

**Table 5:** Intra-observer and Inter-observer reproducibility of STE parameters

	Inter-observer ICC	P value	Intra-observer ICC	P value
EC	0.88 (0.82~ 0.92)	*p* < 0.0001	0.94(0.90~ 0.96)	*p* < 0.0001
ECT	0.91 (0.87~ 0.94)	*p* < 0.0001	0.94(0.92~ 0.96)	*p* < 0.0001
MLS	0.8(0.84~ 0.89)	*p* < 0.0001	0.86(0.83~ 0.89)	*p* < 0.0001
TTO	0.73(0.68~ 0.75)	*p* < 0.0001	0.75(0.73~ 0.77)	*p* < 0.0001
TTP	0.81(0.79~ 0.83)	*p* < 0.0001	0.83(0.81~ 0.85)	*p* < 0.0001
TCT	0.85(0.80~ 0.88)	*p* < 0.0001	0.87(0.83~ 0.90)	*p* < 0.0001
ECR	0.89(0.85~ 0.93)	*p* < 0.0001	0.91(0.87~ 0.95)	*p* < 0.0001
ECRT	0.83(0.80~ 0.90)	*p* < 0.0001	0.86(0.81~ 0.92)	*p* < 0.0001

*EC* effective contraction, *ECT* effective contractile time, *ECR* the ratio of effective contraction, *ECTR* the ratio of effective contractile time, *MLS* maximum longitudinal strain, *TTO* time to onset strain, *TTP* time to peak strain

## Discussion

CRT is a well-established therapy for heart Failure patients and it’s crucial to identify non-responders and responders before CRT and apparently QRS width alone is not enough. Speckle tracking echocardiography (STE) was developed to analyze myocardium deformation and with adapting strain curve, it’s possible to learn the contraction synchrony of different segments and have the advantage of high spatial and temporal resolution and angle independence. Efforts have been made to find the optimal parameters for the detection of CRT responders using STE from different points of view and to better understanding the pathophysiologic mechanism of CRT. Risum et al [26] show that Strain parameters which better reflect a significant LV activation delay, provides an improved tool for dyssynchrony analysis compared to conventional techniques; lumens [27] finds that SSI from strain curves as the sum of posterolateral prestretch percent before aortic valve opening and the septal rebound stretch percent during ejection as a novel parameters to predict CRT, high longitudinal SSI was significantly associated with freedom from the primary endpoint of HF hospitalization or death and secondary endpoint of death. Russel [28, 29] indicates that torsion of the left ventricle (LV) is a promising predictor for CRT. Among patients with severe heart failure, 45 to 75% of patients show rigid body rotation, where the base and apex rotate in the same direction, instead of normal, opposite rotation and the loss of opposite base-apex rotation in patients eligible for CRT is an excellent predictor of acute response and is associated with LV reverse remodeling. Tayal B and Risum et al. [30] report that patients with less dilated left ventricles had nearly fourfold more frequent improvement in dyssynchrony compared to those with severely dilated left ventricles.

Our study used AVO instead of the beginning of QRS as the reference point of time for predicting response to CRT and focused on the mechanical contraction performance in left ventricular ejection period (LV_Ej_), using speckle tracking echocardiography. The current study found that at baseline, the responder group has a more energy-consuming contractile pattern than that of the Non-responder group. At 6-month follow-up, ECR improved significantly in the responder group and contractile efficiency became better than the Non-responder group. Contraction that does not fall into LVEj, either earlier than AVO or later than AVC makes no contribution to cardiac output or LVEF. Myocardial contraction efficiency is closely associated with the CRT response. Compared with the maximum contractility throughout the cardiac cycle (MLS), the contraction during LVEj (EC) provided us the information of contractile efficiency (ECR = EC/MLS*100%). As Galli E [31, 32] analysed ventricular (LV) constructive work (CW) and wasted work (WW) by pressure strain loops (PSLs), and conclude the combination of CW > 1057 mmHg% and WW > 384 mmHg% showed a good specificity (100%) and positive predictive value (100%) but a low sensitivity (22%), negative predictive value (41%), and accuracy (49%) for the identification of CR, though there are different parameters with different analyzing system, it’s still similar with EC and MLS in a prospective of efficiency in cardiac contraction. So it’s important to emphasis the ‘real work’ and ‘real contraction’.

**Fig. 6:**
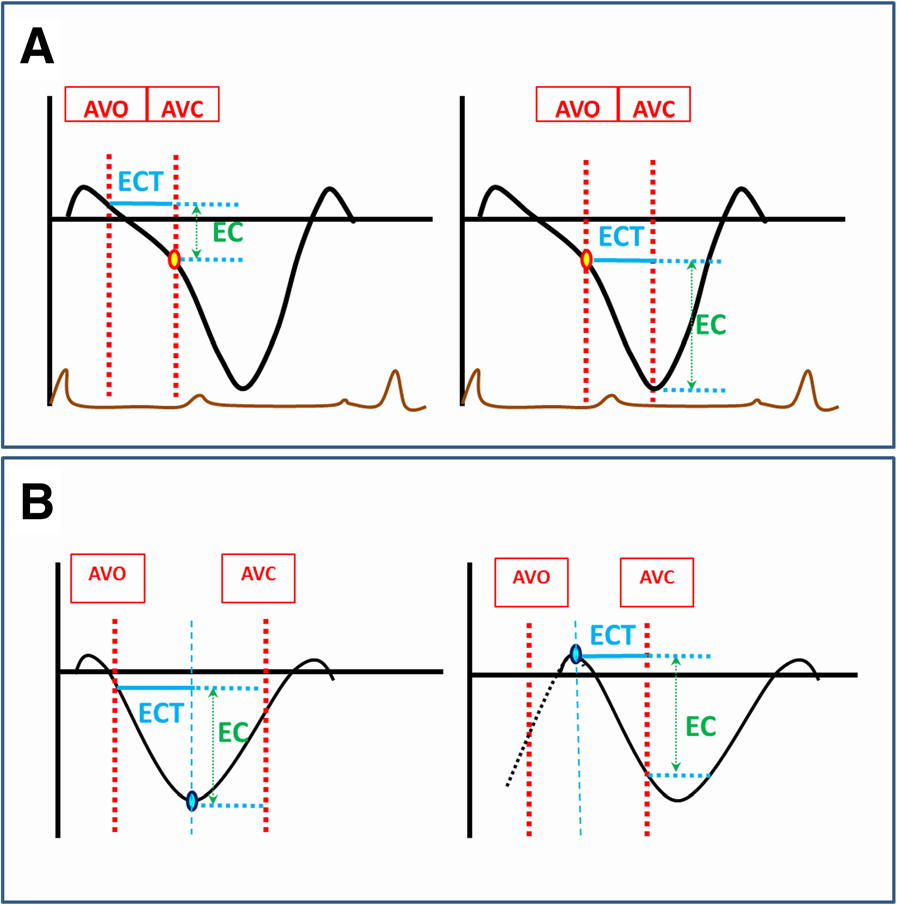
Factors that influence contraction efficiency. **a** shows that when ECT is settled, different contraction velocities (slope) can result in different EC. **b** shows that when contraction velocity is settled, either late onset of contraction after AVO or early end of contraction before AVC may shorten ECT and decrease EC. ECT: effective contractile time; EC: effective contraction; AVC: aortic valve closure; AVO: aortic valve opening

Both contraction velocity and its timing can affect the efficiency (Fig. 6). Compared to the average contraction velocity throughout the whole cardiac cycle, the faster it contracts during the LVEj, the more efficient the contraction pattern will be. It is the same as the time consumption for contraction during LVEj, which is also the more the better. While ECTR indicates only the efficiency related to contraction timing, ECR indicates information of both timing and velocity. Our study indicated that ECR but not ECTR was one of the predictors for CRT response. Russel [31–33] discussed whether mechanical dyssynchrony (regional timing differences) or heterogeneity (regional strain differences) in myocardial function should be used to predict CRT and use the standard deviation (SD) in onset time (T onset) and in time to first peak (T peak, first) of circumferential shortening by Magnetic resonance imaging and find the heterogeneity parameters correlated better with acute response than the mechanical dyssynchrony parameters, so it’s consist with our conclusion of ECR but not ECTR was one of the predictor for CRT response. Besides, Eschalier R, Lumens J et al. [34, 35] show that Left bundle branch block (LBBB) leads to prolonged left ventricular total activation time (TAT) and ventricular electrical uncoupling (VEU; mean LV activation time minus mean right ventricular [RV] activation time); both have been shown to be preferential targets for cardiac resynchronization therapy (CRT), our study show the prolonged TCT at baseline in responder group, and it may can be further explained in the electrophysiology of LBBB.

MLS was the other predictor for CRT which is also a novel index introduced by the current study. It evaluated the maximum length change of myocardium by calculating the difference between positive and negative peak strains rather than the negative peak strain alone to assess the myocardium viability. We found that a higher baseline MLS was associated with more chances of response to CRT. Strain by 2D speckle tracking echocardiography is defined as the percentage of length change of a certain myocardium compared with its original length. Currently, the original length is widely accepted as the length at the beginning of QRS complex on EKG, and the change of length as the difference between the original length and the minimum length before AVC. However, considering the fact that mechanical activation occurs after the electrical activation, we think it is inappropriate to use the onset of electrical activation as the beginning for mechanical deformation. In addition, pre-stretch (PS) and post-systolic shortening (PSS) are often reported in pathologic status such as ischemia and dyssynchrony [35, 36], both of which can lead to the underestimation of contractility by using either systolic peak strain or global peak strain. Therefore, we suggest that MLS, which measures the maximum length difference between the positive peak and the negative peak, should be used to evaluate the viability and the potential of improvement.

In contrast to the previous reports [37], TTP dyssynchrony index was comparable between the Non-responder and the Responder groups while TTO dyssynchrony index was significantly different. As we mentioned, either a TTO later than AVO or a TTP earlier than AVC can increase paradoxical stretch and decrease contraction during LVEj. TTO/TTP dyssychrony indexes themselves cannot predict CRT response. However, they can increase the possibility of TTO after AVO or TTP before AVC and influence the contractile efficiency before CRT. Moreover, although we found significant difference of TTO dyssynchrony index between the Responder and Non-responder group, neither TTO nor TTP dyssynchrony index can predictor CRT response. Therefore, we attributed the importance of TTO/TTP dyssychrony indexes to their possible influence on the contraction time during LVEj.

However, there are some limitations of this study. MLS represents the viability to recover or contract better, ECR has a close relationship with both deformation velocity and time consumption within LVEj, representing the contractile efficiency and the potential to be improved. The current study investigated both important parameters above and regardless of LV lead position, which inevitably influenced CRT response. It may accounts for a suboptimal ROC AUC since it is unknown whether the LV lead is implanted at the most delayed segments and further animal and clinical experiments are needed to verify this. We compared the global strain of each STE parameter while Risum N [26] reports that the reversal in strain-ratio between the early and late-activated myocardial regions at day 1 only among responders suggestive of an important role in promotion of remodeling. Echocardiographic methods for evaluation of mechanical dyssynchrony, reflecting this fundamental pathophysiologic change, could predict response to CRT and provide improved diagnostic value with regards to dyssynchrony analysis compared to conventional TTP measurement, so we may analyze each segment respectively in further study.

## Conclusion

Myocardial contraction pattern within left ventricle ejection indicates the contraction efficiency and has a close relationship with patients’ CRT response. The onset of mechanical activation, rather than the onset of the QRS complex, should be used as the reference point to determine original myocardium length when measuring myocardial contractility. The influence of TTO, TTP, and their dyssynchrony indexes should be carefully interpreted considering their association with LV ejection period.

## Abbreviations

AVC: Aortic valve closure
AVO: Aortic valve opening
CRT: Cardiac resynchronization therapy
EC: Effective contraction
ECG: Electrocardiography
ECR: Ratio of effective contraction
ECT: Effective contractile time
ECTR: Ratio of effective contractile time
LVEF: Left ventricular ejection fraction
LV_Ej_: Left ventricular ejection period
MCE: Myocardial contraction efficiency
MLS: Maximum longitudinal strain
STI: Speckle tracking imaging
TCT: Total contractile time
TDI: Tissue doppler imaging
TTO: Time to the onset of contraction
TTO-16SD: 16-segement standard deviation of time to onset strain
TTP: Time to peak strain
TTP-16SD: 16-segement standard deviation of time to peak strain
